# Getting Closer to a Vaccine for Hookworm

**DOI:** 10.1371/journal.pmed.0020369

**Published:** 2005-10-04

Hookworms are intestinal parasites of mammals, including humans, dogs, and cats; in humans, these infections are a leading cause of intestinal blood loss and iron-deficiency anemia. These infections occur mostly in tropical and subtropical climates, and are estimated to infect about 1 billion people worldwide—about one-fifth of the world's population. People who have direct contact with soil that contains human feces in areas where hookworm is common are at high risk of infection; because children play in dirt and often go barefoot, they are at highest risk.

However, since transmission of hookworm infection requires development of the larvae in soil, hookworm cannot be spread person to person. Anthelminthic chemotherapy with benzimidazole drugs is effective at eliminating existing adult parasites. But since reinfection occurs rapidly after treatment, making a vaccine against hookworm disease is a public health priority. Previous animal vaccine studies have had mixed results. Dogs have been successfully vaccinated against infection with the dog hookworm Ancylostoma caninum by immunization with attenuated third-stage infective larvae (L3). Varying levels of efficacy have been reported for vaccination against the major antigens secreted by the same larval stage in hamsters and dogs. However, only partial reductions in parasite load have been reported. In addition, protective antigens from the larval stage are only expressed in larvae, not in adult worms; hence, antibodies against L3 secretions are useless against adult stage parasites in the gut.

In this month's *PLoS Medicine*, Alex Loukas and colleagues suggest that the ideal hookworm vaccine would be a mixture of two recombinant proteins, targeting both the infective larva and the blood-feeding adult stage of the parasite. Such a vaccine would limit the amount of blood loss caused by feeding worms and maintain normal levels of hemoglobin, said the authors. This outcome is particularly important in young children and women of childbearing age, where menstrual and, particularly, fetal hemoglobin demands are high. [Fig pmed-0020369-g001]

**Figure pmed-0020369-g001:**
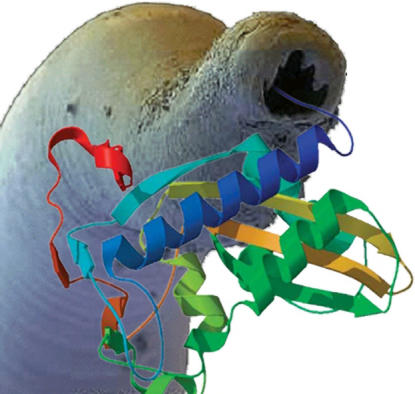
Hookworms secrete proteins that are being used as vaccines in animal models

Of the different proteins expressed by blood-feeding parasitic helminths, proteolytic enzymes have shown promise as intervention targets for vaccine development. A previous study in which dogs were vaccinated with a catalytically active recombinant cysteine hemoglobinase, *Ac*-CP-2, induced antibodies that neutralized proteolytic activity, and provided partial protection to vaccinated dogs by reducing egg output and worm size, but there were not significant reductions of adult worm burdens or blood loss.

In the present study, the researchers found that vaccination of dogs with recombinant *Ac*-APR-1, an aspartic hemoglobinase that initiates the hemoglobin digestion cascade in hookworms, induced antibody and cellular responses, and resulted in significantly reduced hookworm burdens and fecal egg counts in vaccinated dogs compared to control dogs after challenge with infective larvae of A. caninum. Most importantly, vaccinated dogs were protected against blood loss and most did not develop anemia, the major pathologic sequelae of hookworm disease.

The authors went on to show that IgG from vaccinated animals decreased the catalytic activity of the recombinant enzyme in vitro, and the antibody bound in situ to the intestines of worms recovered from vaccinated dogs, implying that the vaccine interfered with the parasite's ability to digest blood.

This result of vaccination against APR-1 shows the best efficacy so far reported for a recombinant vaccine aimed at reducing hookworm egg counts, intestinal worm burdens, and hookworm-induced blood loss, say the authors. They suggest that vaccination with APR-1 damaged the parasite's intestine and resulted in decreased blood intake by feeding worms, and, hence, reduced blood loss from the dogs.

The authors go on to suggest that the optimal hookworm vaccine would combine two elements: one to prevent L3 from developing into adult blood-feeding hookworms, and one to block the establishment, survival, and fecundity of the adult parasites in the intestine. Achieving both goals would require a vaccine comprised of an L3 antigen, such as ASP-2, which is now under clinical development, and an adult gut protease, such as APR-1.

These results have implications for human hookworm vaccine development; the authors finish by saying that there is now enough evidence to conclude that the counterpart vaccine for the major human hookworm Necator americanus (Na-APR-1) should be developed and entered into human clinical trials.

